# Novel Carbon Dots Derived from *Puerariae lobatae* Radix and Their Anti-Gout Effects

**DOI:** 10.3390/molecules24224152

**Published:** 2019-11-16

**Authors:** Xiaoke Wang, Yue Zhang, Meiling Zhang, Hui Kong, Suna Wang, Jinjun Cheng, Huihua Qu, Yan Zhao

**Affiliations:** 1 School of Preclinical Medicine, Beijing Key Laboratory, Beijing University of Chinese Medicine, Beijing 100102, China; wangxiaoke@bucm.edu.cn (X.W.); 18811790361@163.com (M.Z.); doris7629@126.com (H.K.); wsn8212@163.com (S.W.); carlosjjcheng@163.com (J.C.); 2 School of Life Sciences, Beijing University of Chinese Medicine, Beijing 100102, China; 201801024@bucm.edu.cn; 3 Center of Scientific Experiment, Beijing University of Chinese Medicine, Beijing 100102, China; quhuihuadr@163.com

**Keywords:** carbon dots, *Pueraria*, hyperuricemia, uric acid, gouty, new agents

## Abstract

Gout is a disease with a high incidence and causing great harm, and the current treatment drugs are not satisfactory. In this study, novel water-soluble carbon dots (CDs) with anti-gout effect, named *Puerariae lobatae* Radix CDs (PLR-CDs), are reported. PLR-CDs were synthesized with an improved pyrolysis method at 300 °C, and their characterization was performed with multifaceted approaches, such as transmission electron microscopy (TEM) and ultraviolet–visible (UV–vis) and Fourier-transform infrared (FTIR) spectroscopy. In addition, the biocompatibility of PLR-CDs was studied using the cell counting kit (CCK)-8 in LO2 cells and RAW264.7 cells, and the anti-gout activity of PLR-CDs was examined on animal models of hyperuricemia and gouty arthritis. The characterization of PLR-CDs indicated that they were nearly spherical, with diameters ranging from 3.0 to 10.0 nm, and the lattice spacing was 0.283 nm. The toxicity experiment revealed that PLR-CDs were non-poisonous for LO2 cells and RAW264.7 cells at concentrations below 250 μg/mL. The results of pharmacodynamic experiments showed that PLR-CDs could lower the blood uric acid level in model rats by inhibiting the activity of xanthine oxidase and reduce the degree of swelling and pathological damage of gouty arthritis. Thus, PLR-CDs with anti-gout biological activity and good biocompatibility have the prospect of clinical application for the treatment of gout.

## 1. Introduction

Carbon dots (CDs), which are popular nanomaterials with sizes below 10 nm and carrying many functional groups (–OH, –COOH, etc.), have attracted much interest due to their expanded applications [1]. Due to their particular physical and chemical properties, CDs have recently emerged as an important global nanomaterial type [2]. CDs hold a number of advantages, such as excellent water solubility, easy functionalization, resistance to photobleaching, good biocompatibility, and chemical stability [3]. Due to these properties, CDs have applications in biomedical fields, such as drug delivery [4], fluorescent probes for clinical diagnosis [5], bioimaging [6], therapy [7,8], and artificial mimicking enzymes [9]. Recently, more and more intrinsic bioactivities of CDs have been explored and reported, such as homeostatic bioactivity [10,11,12], wound-healing activity [13], hypoglycemic bioactivity [14], hepatoprotective bioactivity [15], radical-scavenging activity [16], peroxidase-like activity [17], anticancer activity [7,18], and antibacterial activity [19,20]. However, as one of the most important nanomaterials, volumes of the studies concerned with CDs’ bioactivities are still small, and the functions of CDs have yet to be fully developed. Presently, the utilization of CDs for the treatment of gout or hyperuricemia has not been reported.

Gout is a pathological condition of purine nucleotide metabolism, in the form of elevated uric acid levels, monosodium urate crystal deposition, and urate-induced inflammatory response around joints [21]. Clinical treatment of gout consists in the rational and long-term reduction of plasma urate concentrations and suppression of inflammation in acute gouty arthritis. Uric acid-lowering drugs, such as xanthine oxidase (XOD) inhibitors, uricosuric agents, and anti-inflammatory drugs, are commonly applied in the clinic for gout treatment, although long-term use of those drugs may cause some side effects, such as bone marrow suppression, gastrointestinal irritation, as well as renal toxicity [22]. Due to the need for blood uric acid control in gout patients [23] and the side effects of clinical drugs, new drugs for gout and hyperuricemia from natural products are urgently needed.

In this work, *Puerariae lobatae* Radix CDs (PLR-CDs), a kind of novel CDs, were synthesized using *P. lobatae* Radix (PLR) as the raw material, with a simple, low-cost, and environmentally friendly pyrolysis method. PLR, which is the dry root of the leguminous plant *P. lobata* (Willd.) Ohwi, is a commonly used Chinese herb for many diseases [24]. Since it contains a large amount of carbon, nitrogen, and oxygen, its biomass is found to be an excellent material for preparing CDs [25,26]. PLR, produced in most Chinese provinces, is a cheap, readily available biomass with environmentally friendly characteristics. After the PLR-CDs were synthesized, their morphology, optical characteristics, and elemental composition were analyzed by various means, such as transmission electron microscopy (TEM) and ultraviolet–visible (UV–vis), Fourier-transform infrared (FTIR), and X-ray photoelectron spectroscopy. Then, we reported the anti-hyperuricemic and anti-inflammatory activities of the obtained PLR-CDs.

## 2. Results

### 2.1. Characterization of PLR-CDs

**Figure 1:**
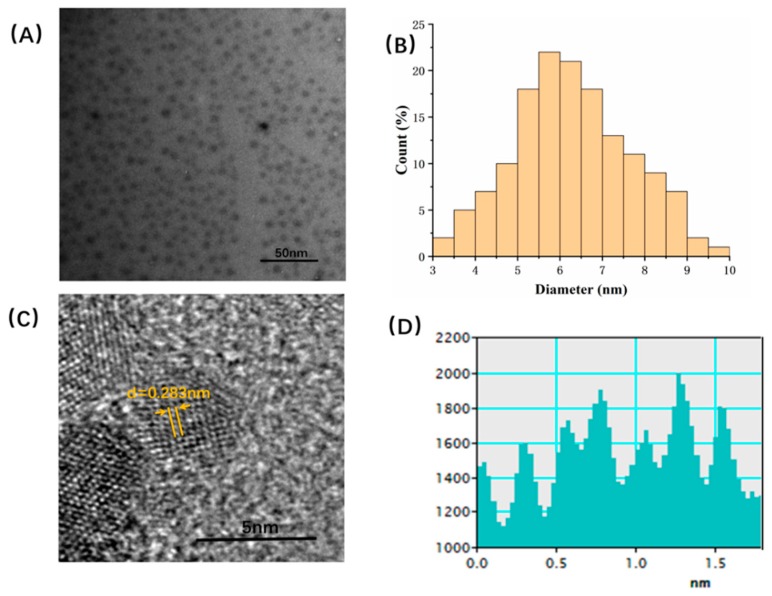
Characterization of *Pueraria* carbon dots. (**A**) Transmission electron microscopy (TEM) images of *Puerariae lobatae* Radix carbon dots (PLR-CDs). (**B**) TEM size distribution of PLR-CDs. (**C**) High-resolution TEM (HRTEM) image of PLR-CDs. (**D**) Line profiles of the corresponding HRTEM images of PLR-CDs analyzed on HRTEM.

The TEM image of the PLR-CDs in Figure 1A shows that the CDs were spherical and had different sizes. The dispersibility of PLR-CDs was good, and no accumulation was observed. The diameter of the CDs was in a narrow range: 3–10 nm (Figure 1B). Additionally, high-resolution TEM (HRTEM) revealed that the lattice spacing of the CDs was 0.283 nm (Figure 1C).

**Figure 2:**
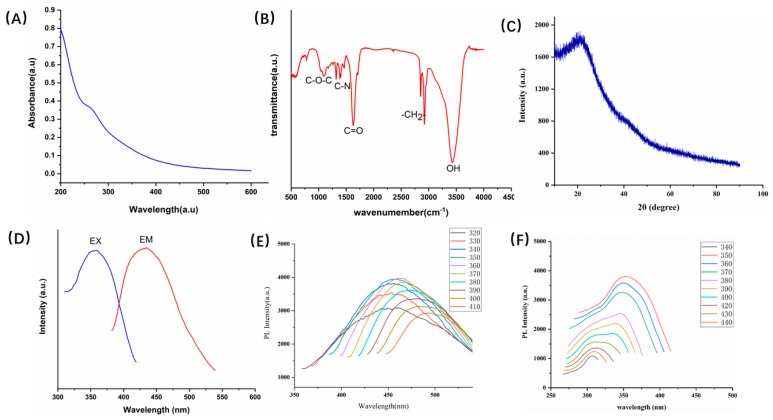
(**A**) Ultraviolet-visible spectrum of *Pueraria* carbon dots (PLR-CDs). (**B**) Fourier-transform infrared spectrum of PLR-CDs. (**C**) XRD pattern of the PLR-CDs. (**D**) Fluorescence spectra of PLR-CDs, EM represents emission spectra, EX represents excitation spectra. (**E**) Fluorescence spectra of PLR-CDs at different excitation wavelengths. (**F**) Fluorescence spectra of PLR-CDs at different excitation wavelengths.

The optical properties were studied by UV–vis, and the absorption spectrum revealed a weak adsorption peak at 271 nm for the PLR-CDs solution, which was attributed to the π–π* transition of the C=C bond. In addition, FTIR was used to investigate the CDs for a better understanding of the organic functional groups on their surfaces, and the purified PLR-CDs spectra (Figure 2B) showed characteristic peaks at 3434, 2921, 2853, 1630, 1384, and 1090 cm^−1^. The presence of O–H groups was indicated by the peak at 3434 cm^−1^. A characteristic absorption peak related to the C–N band was observed at 1630 cm^−1^. Besides, the presence of weak absorption at 2921 and 2853 cm^−1^ indicated C–H stretching, while the peaks at 1384 cm^−1^ and 1090 cm^−1^ were representative of the C–N band and weak C–O stretching band, respectively. In addition, the XRD pattern shown in Figure 2C demonstrates a distinct diffraction peak (2θ = 21.3), which was attributed to amorphous carbon composed in a considerably random fashion [10]. The emission spectra were observed with the maximum emission at 454 nm under the maximum excitation of 355 nm (Figure 2D). By reference to quinine sulphate, the quantum yield (QY) of the PLR-CDs was calculated under the excitation of 355 nm, and it was calculated to be 3.2%. In order to further explore the optical properties of the as-synthesized PLR-CDs, the maximum excitation wavelength was investigated under different emission wavelengths. When the emission wavelength was changed from 420 to 490 nm, the maximum excitation wavelengths were determined to be 330 to 373 nm. The photoluminescence behavior of PLR-CDs under excitation from 320 to 420 nm was investigated and is displayed in Figure 2E, in which the maximum fluorescent intensity was recorded at 340 nm excitation wavelength.

**Figure 3:**
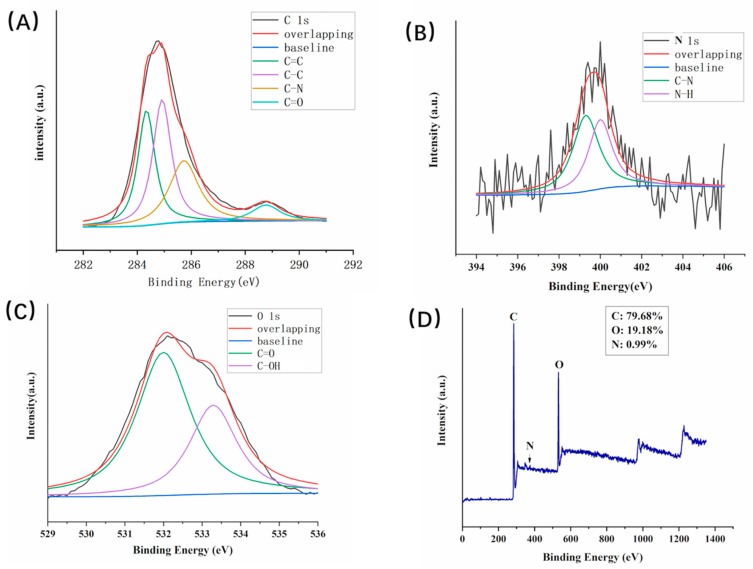
Surface composition and elemental analysis of the prepared PLR-CDs determined by XPS. High-resolution XPS spectra of (**A**) C 1s, (**B**) O 1s, and (**C**) N 1s peaks of the PLR-CDs. (**D**) Full-scan XPS spectrum of PLR-CDs.

The XPS technique was used to analyze the element composition as well as the surface groups of the prepared PLR-CDs. As shown in Figure 3, the synthesized CDs were mainly composed of the elements C 1s and O 1s at relative percentage compositions of 79.68% and 19.18%, respectively. In addition, the elements N 1s (0.99%) and S 2p (0.16%) also existed in CDs. The elements C, O, and N may correspond to C–C, C–OH, COOH, C–N, and C–O–C. Moreover, no heavy metal elements were detected. The high-resolution XPS spectrum of C 1s in Figure 3A can be divided into four peaks with binding energies at 284.3, 284.9, 285.7, and 288.8 eV, corresponding to sp^2^ C, C–C, C–N, and C=O bonds, respectively. The O 1s spectrum in Figure 3C can be divided into two peaks at 532.0 and 533.3 eV, which correspond to C=O and C–OH, respectively. The N 1s spectrum (Figure 3B) can be fitted with two peaks at 400 and 399.3 eV, which were attributed to N–H and C–N, respectively. This result is in accordance with the surface composition of PLR-CDs shown in the FTIR analysis.

### 2.2. Cellular Toxicity

**Figure 4:**
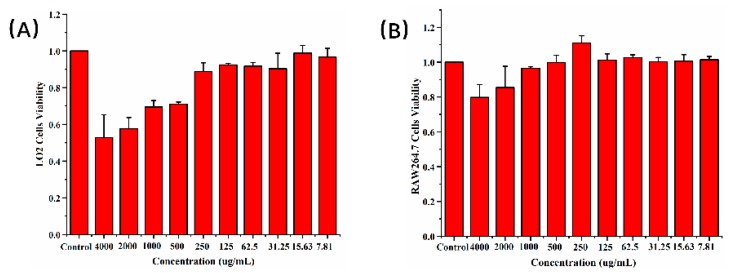
Cell viability results of (**A**) LO2 and (**B**) RAW264.7 after incubation with free PLR-CDs (control) at different concentrations ranging from 7.81 to 4000 μg/mL for 24 h.

To verify the biocompatibility of CDs, cellular toxicity was evaluated on LO2 cells and RAW264.7 cells using the cell counting kit (CCK)-8 cell viability assay. Figure 4A shows that the cell viability of LO2 cells cocultured with PLR-CDs (7.81–250 μg/mL) did not decrease after 24 h of incubation. However, when the concentration of PLR-CDs increased from 500 to 4000 μg/mL, the cell viability of LO2 cells began to decrease. The minimum cell viability was 52% at 4000 μg/mL. For RAW264.7 cells, PLR-CDs at concentrations from 7.81 to 1000 μg/mL did not reduce cell viability, whereas PLR-CDs at a concentration of 250 μg/mL increased cell viability to 111%. Cell viability was slightly reduced to 85% and 80%, respectively, as the concentrations of PLR-CDs increased to 2000 and 4000 μg/mL. This shows that the cytotoxicity in LO2 cells was negligible when the concentration of PLR-CDs was lower than 250 μg/mL, and the cytotoxicity in RAW264.7 cells was negligible when when the concentration of PLR-CDs was lower than 1000 μg/mL. We can conclude that PLR-CDs have good biocompatibility.

### 2.3. Anti-Hyperuricemic Bioactivity and Effect on XOD of PLR-CDs

#### 2.3.1. Anti-Hyperuricemic Bioactivity of PLR-CDs

**Figure 5:**
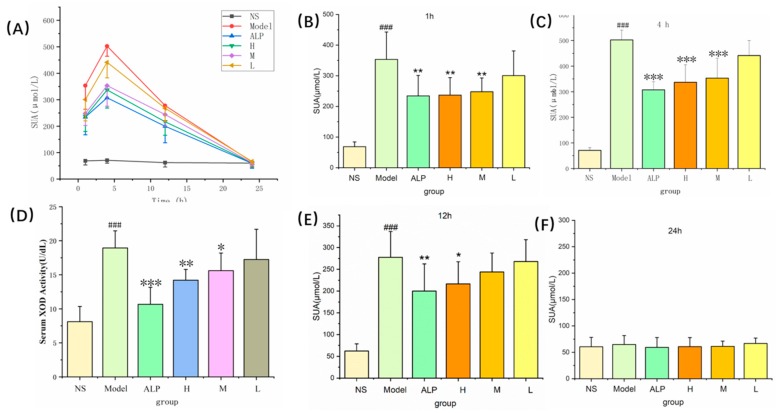
Effects of PLR-CDs on serum uric acid levels for 24 h (**A**) and serum xanthine oxidase (XOD) activity for 1 h (**D**) in hyperuricemic rats. NS and Model represent normal control group and model group, respectively; ALP, H, M, and L indicate rats treated with allopurinol and high, medium, and low doses, respectively, of PLR-CDs. Serum uric acid level was measured at 1 h (**B**), 4 h (**C**), 12 h (**D**), and 24 h (**E**). ^#^ *p* < 0.05, ^##^ *p* < 0.01, ^###^ *p* < 0.001 when compared to NS group; ∗ *p* < 0.05, ∗∗ *p* < 0.01, ∗∗∗ *p* < 0.001 when compared to the ALP group.

To evaluate the hypouric acid activity of PLR-CDs and their effect on XOD activity, the rat model of hyperuricemia induced by oxonate and hypoxanthine was used. In the plasma uric acid test, the uric acid levels of the model group, allopurinol-treated group, and PLR-CDs-treated group were significantly increased from 1 h to 12 h, compared with that of the normal control group (NS group). However, the uric acid levels in rats treated with three doses of PLR-CDs were lower than those in the model group from 1 h to 12 h. At 24 h, the uric acid levels in each group returned to normal, and there was no statistically significant difference between the six groups (Figure 5A).

At 1 h, the uric acid levels of the 4 mg/kg PLR-CDs-treated group (236 ± 57 μmol/L) and 2 mg/kg PLR-CDs-treated group (247 ± 45 μmol/L) were significantly lower (*p* < 0.01) than those of the model group (353 ± 89 μmol/L) (Figure 5B).

At 4 h, the uric acid levels of the 4 mg/kg PLR-CDs-treated group (336 ± 68 μmol/L) and 2 mg/kg PLR-CDs-treated group (353 ± 78 μmol/L) were significantly lower (*p* < 0.01) than those of the model group (502 ± 38 μmol/L) (Figure 5C).

At 12 h, the uric acid levels of the 4 mg/kg and 2 mg/kg PLR-CDs-treated groups were 216 ± 43 μmol/L, *p* < 0.01, which were lower than those of the model group (277 ± 59 μmol/L) (Figure 5E).

#### 2.3.2. Effect on XOD of PLR-CDs

To study the mechanism by which PLR-CDs reduce uric acid, the blood taken at 1 h was also used to determine the activity of XOD. Figure 5D shows that the plasma XOD activity of the model group was significantly enhanced (19 ± 3 U/dL) compared with that of the NS group (8 ± 2 U/dL) (*p* < 0.01). The XOD activity of the groups treated with high, medium, and low doses of PLR-CDs were 14 ± 2 U/dL, 16 ± 3 U/dL, and 17 ± 4 U/dL, respectively. In addition, compared with the model group, PLR-CDs at 4 and 2 mg/kg remarkably suppressed plasma XOD activity with statistical significance.

### 2.4. Effect of PLR-CDs in Rat Models of Monosodium Urate (MSU)-Induced Arthritis

**Figure 6:**
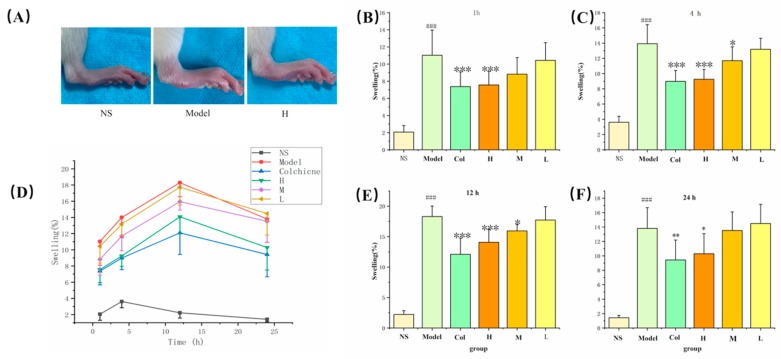
Representative images of joints from NS, Model, and H groups at 12 h (**A**). Effects of PLR-CDs on the swelling degree of rats’ joints after molding for 24 h (**D**). NS and Model represent normal control group and model group, respectively; Col, H, M, and L were treated with colchicine and high, medium, and low doses, respectively, of PLR-CDs. The swelling degree was measured and calculated at 1 h (**B**), 4 h (**C**), 12 h (**E**), and 24 h (**F**).

As shown in Figure 6, the model of gouty arthritis was established successfully, for the swelling degree of ankles in the model group was significantly higher than that of the NS group. The swelling level in the normal control group was always stable at an almost horizontal level during the entire experiment. After 12 h after MSU treatment, the degree of joint swelling reached its maximum value, then gradually declined. PLR-CDs minimized the ankle swelling from 1 h to 24 h, showing a similar effect to that of the positive drug. Of note, the swelling levels of the H group (4 mg/kg) were significantly lower than those of the model group at 1 h (*p* < 0.001), 4h (*p* < 0.001), and 12h (*p* < 0.05), and the M group also induced swelling at 4 h and 12 h.

**Figure 7:**
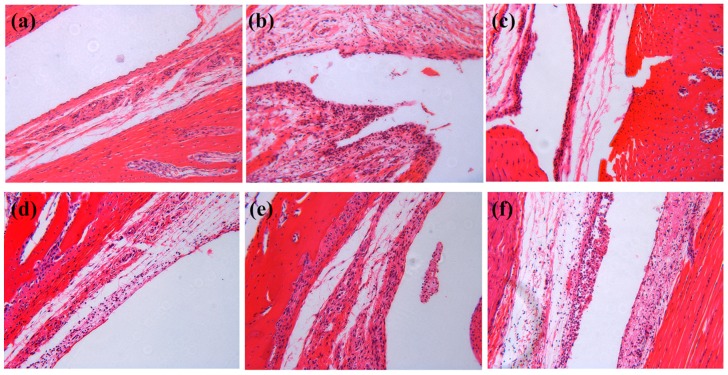
Histological examination of H and E stained ankle joint tissues of normal group (**a**), model group (**b**), Col, H, M, and L treated group (**c**–**f**).

In order to evaluate the severity of inflammation in the ankle joints, joint tissues were collected and examined by hematoxylin and eosin (H&E)staining. As shown in Figure 7, histological changes such as synovial hyperplasia and extensive infiltration of inflammatory cells in the synovial membrane could be observed in the model group, whereas the severity of inflammatory cell infiltration, synovial hyperplasia, and edema was alleviated in colchicine- and PLR-CDs-treated groups. The results showed that PLR-CDs could play a therapeutic role similar to colchicine, i.e., to alleviate the pathological damage of MSU-induced gouty arthritis.

## 3. Discussion

CDs have been a research hotspot, and a lot of research on their synthetic methods, raw materials, and applications has been performed. By changing the size, shape, composition, and surface chemistry of nanomaterials, their properties can be flexibly regulated, which enables them to be deeply researched and applied in many fields, such as biosensing and therapy. Despite these major achievements, problems such as production costs and environmental toxicity have followed. In order to obtain safe and effective target products, researchers have used various strategies, such as replacing inorganic materials with organic ones in the production process and adopting biosynthesis technology [27,28,29,30]. Impressively, in this paper, biomass was used as the major material, and no metal sites, strong solvents, or strong acids were imported or used during the synthetic process, which indicates that the improved synthetic method used is environmentally friendlier, greener, and safer than previously reported methods. It should be noted that using bioproducts as raw materials has also disadvantages. Although the PLR, purchased from a medicinal materials company, met the standards required by Chinese Pharmacopoeia, using biomass (PLR) as a precursor for CDs synthesis still increases the uncontrollable factors, because the components of biomass vary with the place of origin and collection time of the biomaterial as well as with many other factors, affecting the consistency of the final products.

The pharmacological experiments showed that PLR-CDs exhibit similar hypouricemic potency to allopurinol. Since XOR is a key enzyme that catalyzes the oxidation of subxanthine to xanthine, which is closely related to the excessive production of uric acid, the activities of XOD were tested, and the results demonstrated that PLR-CDs reduced uric acid levels by inhibiting XOD.

### Primary Causes

Hyperuricemia and uric acid-induced inflammatory reactions are the primary causes of gout. Therefore, controlling uric acid levels and inhibiting acute inflammation are two important strategies for the treatment of gout. However, there is no drug that combines both traits. For instance, allopurinol, an XOD inhibitor, can successfully control uric acid levels but fails to reduce inflammation in the acute phase of gout. It is noteworthy that PLR-CDs also exhibited anti-inflammatory activities by improving the ankle swelling and synovial inflammatory injury in rats with acute gouty arthritis. In addition, clinically used therapeutic drugs such as allopurinol and indomethacin have side effects [31,32], so does colchicine [33]. As a result, researchers have been looking for the development of safer and more powerful new drugs with both anti-hyperuricemic and anti-inflammatory effects. Therefore, PLR-CD may become a supplemental and alternative treatment for hyperuricemia and gout.

This study is a preliminary assessment of the anti-hyperuricemia and anti-inflammatory activity, as well as the mechanisms of action, of PLR-CDs. The detailed potential mechanisms of these effects should be elucidated in future studies.

As a popular topic in modern science, CDs are highly biocompatible and are more likely to be therapeutic agents than other nanomaterials. Actually, studies related to the biological activity and mechanism of CDs are less reported than those examining CDs for drug delivery, imaging, and environmental protection. In this paper, PLR-CDs exhibited anti-gout effect by reducing the level of uric acid in the blood and relieving inflammation around the ankles in model animals, which help broaden our understanding of the biological activities of CDs and lay the foundation for future drug discovery.

## 4. Materials and Methods

### 4.1. Chemicals

The PLR was purchased from the Beijing Qiancao Herbal Pieces Co., Ltd. (Beijing, China), and the PLR carbonisatum was prepared in our laboratory. Hypoxanthine (HX, 99%), potassium oxonate (PO, 98%), allopurinol (ALP, 99.5%), and monosodium urate (SMU, standard agent) were purchased from Shanghai Yuanye Bio-Technology Co., Ltd., (Shanghai, China). Colchicine (COL, standard agent) was purchased from Beijing BioDee Biotech Co., Ltd. (Beijing, China). The cell counting kit (CCK)-8 was purchased from Dojindo Molecular Technologies, Inc. (Kumamoto, Japan). Pentobarbital sodium and other analytical-grade chemical reagents were obtained from Sinopharm Chemical Reagents Beijing (Beijing, China). Dialysis membranes with a pore size for a molecular weight of 1000 Da were purchased from Beijing Ruida Henghui Technology Development Co., Ltd. (Beijing, China). All the experiments were performed using deionized water.

### 4.2. Preparation of PLR-CDs

**Figure 8:**
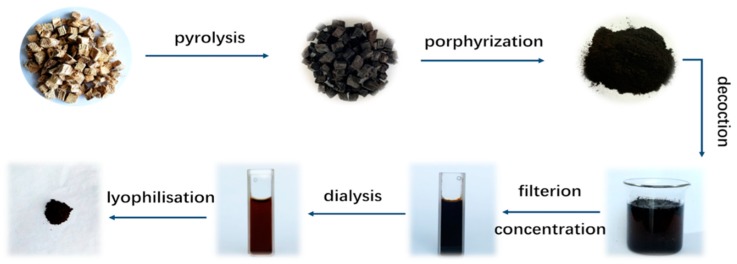
Flow chart of the *Pueraria* carbon dots (PLR-CDs) preparation process.

The PLR-CDs were prepared in a one-step eco-friendly manner, as reported by Liu [12]. The PLR was placed in a crucible sealed by a foil paper, and then the crucible was calcined in a muffle furnace (TL0612, Beijing Zhong Ke Aobo Technology Co., Ltd., Beijing, China) at 300 °C for 1 h. The PLR carbonisatum was finally crushed into a fine powder. Deionized water (DW)was added to the powder (1:20), which boiled three times, one hour each time. The filter membrane suction method and vacuum rotary evaporation were used for removing residue and for concentratating the sample, and the obtained solution was dialyzed against DW for 1 week using a dialysis membrane with a molecular weight cut-off of 1000 Da to get the purified PLR-CDs. The PLR-CDs solution was freeze-dried using a vacuum freeze-drying method in a freeze-dryer (LGJ-10C, Four-ring Science Instrument Plant Beijing Co., Ltd, Beijing, China), and then the PLR-CDs powder was collected and diluted to 0.8, 0.4, and 0.2 mg/mL (high, medium, and low dose, respectively). The preparation of PLR-CDs is shown in Figure 8.

### 4.3. Characterization of PLR-CDs

The morphology, atomic lattice fringes, as well as other microscopic features of PLR-CDs were studied with the TEM (Tecnai G2 20, FEI Company, Hillsboro, OR, USA) and HRTEM systems (JEN-1230, Japan Electron Optics Laboratory, Tokyo, Japan). The spectroscopic studies of the PLR-CDs were performed by fluorescence spectroscopy (F-4500, Tokyo, Japan) and UV-vis spectroscopy (CECIL, Cambridge, UK). Furthermore, FTIR spectra (Thermo Fisher, Fremont, CA, USA) were collected to analyze the functional organic groups in the product. The XPS (ESCALAB 250Xi, Thermo Fisher Scientific, Fremont, CA, USA) measurement was obtained for elemental analysis and surface composition. The XRD (D8-Advanced X-ray diffractometer, Bruker AXS, Karlsruhe, Germany) was performed with Cu K-alpha radiation (λ = 1.5418 Å).

### 4.4. Animals

Male SD rats (190.0–210.0 g) were purchased from Si Beifu (Beijing) Biotechnology Co., Ltd. and maintained under controlled conditions of 12 h light/dark cycle, at 24.0 ± 1.0 °C and 55.0 ± 15.0% humidity. All experimental procedures were approved by the Committee of Ethics of Animal Experimentation of the Beijing University of Traditional Chinese Medicine (BUCM-4-2018101601-4006).

### 4.5. In Vitro Cell Viability in the Presence of PLR-CDs

LO2 cells and RAW264.7 cells were seeded in 96-well plates and allowed to attach for 24 h. Then, the cells were treated with various concentrations of PLR-CDs (4000, 2000, 1000, 500, 250, 125, 62.5, 31.25, 15.63, 7.81, and 0 μg/mL) for 24 h. The cell viability was measured according to the instructions provided in the CCK-8.

### 4.6. Effect of PLR-CDs on Hyperuricemic Rats

The SD rats were divided evenly into six groups (n = 6). According to an improved method reported before [34], potassium oxonate (300 mg/kg, by intraperitoneal injection) and hypoxanthine (500 mg/kg, by intragastric administration) were administered to the rats to an establish acute hyperuricemia model. After an hour, hyperuricemic rats in the model group were treated with normal saline, and other hyperuricemic rats were treated with 5 mg/kg allopurinol (positive control drugs; ALP group) and high, medium, and low doses of PLR-CDs (4, 2, and 1 mg/kg; H-, M-, and L-group, respectively) by intragastric administration. Then, 1.5 mL of blood was collected in EDTA-K2 tubes 1, 4, 12, and 24 h after therapeutic drugs administration. Plasma was isolated bycentrifugation (3500 r/min, 10 min). The levels of uric acid in plasma were tested by a biochemical auto analyzer (AU480, Beckman Coulter, lnc., Brea, California, USA). The activity of xanthine oxidase (XOD) in the plasma were determined using an XOD kit (Solarbio, Beijing, China).

### 4.7. Effect of PLR-CDs on Gouty Arthritis

Using the same method described above, 36 rats were divided and pretreated (the drug in the positive control group was replaced by colchicine). The rats were anesthetized 1 h after pretreatment, using sodium pentobarbital. The NS group (used as a normal control group) received an injection of sterile saline (0.2 mL) into the articular cavity of the right hind foot, while the other groups were injected with an MSU crystal suspension (1 mg in 50 μL of sterile saline) [35,36]. The circumference of swelling ankle joints was measured using a vernier caliper before injection, and 1 h, 4 h, 12 h, and 24 h after injection with the MSU crystal suspension, and the degree of swelling of the joints was calculated with the formula below:

(1) $$D=\frac{Cb-Ca}{Cb}\times 100\%$$

Cb represents the ankle circumference (mm) before model establishment, and Ca represents the ankle circumference (mm) after model establishment.

The animals were sacrificed by cervical dislocation after 24 h, and the ankle joint was removed and fixed in 10% formalin, decalcified using 10% ethylenediaminetetraacetic acid (EDTA), embedded in paraffin (60 °C), cut into sections (4 μm slices), and stained with hematoxylin and eosin. The tissues were observed under a microscope (Olympus IX73P1F Tokyo, Japan), photographed, and processed using a charge-coupled device (Qimaging Q41305, Surrey, British Columbia, Canada) and imaging software (Q-Capture Pro 7.lik).

### 4.8. Statistical Analysis

All of the data were expressed as mean ± standard deviation. Differences between the groups were determined by one-way analysis of variance (ANOVA), followed by the least significant difference test (LSD), and values of *p* < 0.05 were considered significant. Statistical analyses were carried out using SPSS software, version 24.0, (SPSS Inc., Chicago, IL, USA).

## 5. Conclusions

In this work, PLR-CDs, a kind of multifunctional carbon dots, were synthesized via a one-step environmentally friendly process using PLR as the precursor. The obtained PLR-CDs with a QY of 3.2% were characterized using TEM, HRTEM, and UV–vis fluorescence, and FTIR spectroscopy, as well as XPS and XRD. Further pharmacodynamic experiments in potassium oxonate–hypoxanthine-induced hyperuricemia rats revealed the hypouricemic effects of the PLR-CDs, and the hypouricemic efficacy was exerted primarily through inhibition of xanthine oxidase activity. PLR-CDs showed anti-inflammatory activity, manifested as reduction of the ankle swelling degree and alleviation of pathologic damage in model animals with gouty arthritis. The present study gives valuable biophysical insight into the anti-hyperuricemic and anti-inflammatory activities of PLR-CDs, which might contribute to facilitating the development of potential new treatment strategies for gout and hyperuricemia. Moreover, it provides a novel strategy for exploring the biological effects of the materials employed in traditional Chinese medicine.

## References

[1] Jia W., Tang B., Wu P. (2018). Carbon Dots with Multi-Functional Groups and the Application in Proton Exchange Membranes. Electrochim. Acta.

[2] Atabaev T.S. (2018). Doped Carbon Dots for Sensing and Bioimaging Applications: A Minireview. Nanomaterials.

[3] Zhou N., Hao Z., Zhao X., Maharjan S., Zhu S., Song Y., Yang B., Lu L. (2015). A Novel Fluorescent Retrograde Neural Tracer: Cholera Toxin B Conjugated Carbon Dots. Nanoscale.

[4] Ding H., Du F., Liu P., Chen Z., Shen J. (2015). DNA–Carbon Dots Function as Fluorescent Vehicles for Drug Delivery. ACS Appl. Mater. Interfaces.

[5] Yu Y., Yang Y., Ding J., Meng S., Li C., Yin X. (2018). Design of a Biocompatible and Ratiometric Fluorescent Probe for the Capture, Detection, Release, and Reculture of Rare Number CTCs. Anal. Chem..

[6] Sri S., Kumar R., Panda A.K., Solanki P.R., Panda D.A. (2018). Highly Biocompatible, Fluorescence, and Zwitterionic Carbon Dots as a Novel Approach for Bioimaging Applications in Cancerous Cells. ACS Appl. Mater. Interfaces.

[7] Prasad R., Chauhan D.S., Yadav A.S., Devrukhkar J., Singh B., Gorain M., Temgire M., Bellare J., Kundu G.C., Srivastava R. (2018). A Biodegradable Fluorescent Nanohybrid for Photo-Driven tumor Diagnosis and Tumor Growth Inhibition. Nanoscale.

[8] Xue M., Zhao J., Zhan Z., Zhao S., Lan C., Ye F., Liang H. (2018). Dual Functionalized Natural Biomass Carbon Dots from Lychee Exocarp for Cancer Cell Targetable Near-Infrared Fluorescence Imaging and Photodynamic Therapy. Nanoscale.

[9] Lv Y., Ma M., Huang Y., Xia Y. (2019). Carbon Dot Nanozymes: How to Be Close to Natural Enzymes. Chem. A Eur. J..

[10] Sun Z., Lu F., Cheng J., Zhang M., Zhang Y., Xiong W., Zhao Y., Qu H. (2018). Haemostatic Bioactivity of Novel Schizonepetae Spica Carbonisata-Derived Carbon Dots Via Platelet Counts Elevation. Artif. Cells Nanomed. Biotechnol..

[11] Yan X., Zhao Y., Luo J., Xiong W., Liu X., Cheng J., Wang Y., Zhang M., Qu H. (2017). Hemostatic Bioactivity of Novel Pollen Typhae Carbonisata-Derived Carbon Quantum Dots. J. Nanobiotechnol..

[12] Liu X., Wang Y., Yan X., Zhang M., Zhang Y., Cheng J., Lu F., Qu H., Wang Q., Zhao Y. (2018). Novel Phellodendri Cortex (Huang Bo)-Derived Carbon Dots and Their Hemostatic Effect. Nanomedicine.

[13] Chatzimitakos T.G., Kasouni A.I., Troganis A.N., Stalikas C.D. (2018). Carbonization of Human Fingernails: Toward the Sustainable Production of Multifunctional Nitrogen and Sulfur Codoped Carbon Nanodots with Highly Luminescent Probing and Cell Proliferative/Migration Properties. ACS Appl. Mater. Interfaces.

[14] Sun Z., Lu F., Cheng J., Zhang M., Zhu Y., Zhang Y., Kong H., Qu H., Zhao Y. (2018). Hypoglycemic Bioactivity of Novel Eco-Friendly Carbon Dots Derived from Traditional Chinese Medicine. J. Biomed. Nanotechnol..

[15] Cheng J., Zhang M., Sun Z., Lu F., Xiong W., Luo J., Kong H., Wang Q., Qu H., Zhao Y. (2019). Hemostatic and Hepatoprotective Bioactivity of Junci Medulla Carbonisata-Derived Carbon Dots. Nanomedicine.

[16] Zhao S., Lan M., Zhu X., Xue H., Ng T.W., Meng X., Lee C.S., Wang P.F., Zhang W. (2015). Green Synthesis of Bifunctional Fluorescent Carbon Dots from Garlic for Cellular Imaging and Free Radical Scavenging. ACS Appl. Mater. Interfaces.

[17] Shi W., Wang Q., Long Y., Cheng Z., Chen S., Zheng H., Huang Y. (2011). Carbon Nanodots as Peroxidase Mimetics and Their Applications to Glucose Detection. Chem. Commun..

[18] Yao H., Li J., Song Y., Zhao H., Wei Z., Li X., Jin Y.R., Yang B., Jiang J. (2018). Synthesis of Ginsenoside Re-Based Carbon Dots Applied for Bioimaging and Effective Inhibition of Cancer Cells. Int. J. Nanomed..

[19] Zhang J., Liu X., Wang X., Mu L., Yuan M., Liu B., Shi H. (2018). Carbon Dots-Decorated Na2W4O13 Composite with WO3 for Highly Efficient Photocatalytic Antibacterial Activity. J. Hazard. Mater..

[20] Bing W., Sun H., Yan Z., Ren J., Qu X. (2016). Programmed Bacteria Death Induced by Carbon Dots with Different Surface Charge. Small.

[21] Perez Ruiz F., Dalbeth N. (2019). Combination Urate-Lowering Therapy in the Treatment of Gout: What is the Evidence?. Semin. Arthritis Rheum..

[22] Liang G., Nie Y., Chang Y., Zeng S., Liang C., Zheng X., Xiao D., Zhan S.Q., Zheng Q. (2019). Protective Effects of Rhizoma Smilacis Glabrae Extracts on Potassium Oxonate-And Monosodium Urate-Induced Hyperuricemia and Gout in Mice. Phytomedicine.

[23] Cui L., Meng L., Wang G., Yuan X., Li Z., Mu R., Wu S. (2017). Prevalence and Risk Factors of Hyperuricemia: Results of the Kailuan Cohort Study. Mod. Rheumatol..

[24] Wu Y., Wang X., Fan E. (2012). Optimisation of Ultrasound-Assisted Extraction of Puerarin and Total Isoflavones from Puerariae Lobatae Radix (Pueraria Lobata (Wild.) Ohwi) with Response Surface Methodology. Phytochem. Anal..

[25] Miao H., Wang L., Zhuo Y., Zhou Z., Yang X. (2016). Label-Free Fluorimetric Detection of CEA Using Carbon Dots Derived from Tomato Juice. Biosens. Bioelectron..

[26] Vandarkuzhali S.A.A., Jeyalakshmi V., Sivaraman G., Singaravadivel S., Krishnamurthy K.R., Viswanathan B. (2017). Highly Fluorescent Carbon Dots from Pseudo-Stem of Banana Plant: Applications as Nanosensor and Bio-Imaging Agents. Sens. Actuators B Chem..

[27] Hasan M., Ullah I., Zulfiqar H., Naeem K., Iqbal A., Gul H., Ashfaq M., Mahmood N. (2018). Muhammad Ashfaq Biological Entities as Chemical Reactors for Synthesis of Nanomaterials: Progress, Challenges and Future Perspective. Mater. Today Chem..

[28] Yousaf M., Ahmad M., Bhatti I.A., Nasir A., Hasan M., Jian X., Kalantar Zadeh K., Mahmood N. (2019). In Vivo and In Vitro Monitoring of Amyloid Aggregation via BSA@FGQDs Multimodal Probe. Acs Sens..

[29] Hasan M., Zafar A., Yousaf M., Gulzar H., Mehmood K., Hassan S.G., Saeed A., Yousaf A., Mazher A., Rongji D. (2019). Synthesis of Loureirin B-Loaded Nanoliposomes for Pharmacokinetics in Rat Plasma. ACS Omega.

[30] Zulfiqar H., Zafar A., Rasheed M.N., Ali Z., Mehmood K., Mazher A., Hasan M., Mahmood N. (2019). Synthesis of Silver Nanoparticles Using Fagonia Cretica and Their Antimicrobial Activities. Nanoscale Adv..

[31] Wang C.W., Dao R.L., Chung W.H. (2016). Immunopathogenesis and Risk Factors for Allopurinol Severe Cutaneous Adverse Reactions. Curr. Opin. Allergy Clin. Immunol..

[32] Tziona P., Theodosis Nobelos P., Rekka E. (2017). Medicinal Chemistry Approaches of Controlling Gastrointestinal Side Effects of Non-Steroidal Anti-Inflammatory Drugs. Endogenous Protective Mechanisms and Drug Design. Med. Chem..

[33] Tong D.C., Wilson A.M., Layland J. (2016). Colchicine in Cardiovascular Disease: An Ancient Drug with Modern Tricks. Heart.

[34] Yong T., Zhang M., Chen D., Shuai O., Chen S., Su J., Jiao C., Feng D., Xie Y. (2016). Actions of Water Extract from Cordyceps Militaris in Hyperuricemic Mice Induced by Potassium Oxonate Combined with Hypoxanthine. J. Ethnopharmacol..

[35] Coderre T.J., Wall P.D. (1987). Ankle Joint Urate Arthritis (AJUA) in Rats: An Alternative Animal Model of Arthritis to That Produced by Freund’s Adjuvant. Pain.

[36] Coderre T.J., Wall P.D. (1988). Ankle Joint Urate Arthritis in Rats Provides a Useful Tool for the Evaluation of Analgesic and Anti-Arthritic Agents. Pharmacol. Biochem. Behav..

